# Comparison of Hemodynamic Support by Impella vs. Peripheral Extra-Corporeal Membrane Oxygenation: A Porcine Model of Acute Myocardial Infarction

**DOI:** 10.3389/fcvm.2020.00099

**Published:** 2020-06-10

**Authors:** Christoph Nix, Kiyotake Ishikawa, Bart Meyns, Shota Yasuda, Tom Adriaenssens, Svenja Barth, Rashad Zayat, Pascal Leprince, Guillaume Lebreton

**Affiliations:** ^1^Department of Anesthesiology, RWTH Aachen University Hospital, Aachen, Germany; ^2^Abiomed Europe GmbH, Aachen, Germany; ^3^Icahn School of Medicine at Mount Sinai, New York, NY, United States; ^4^Department of Cardiac Surgery, University Hospital UZ Leuven, Leuven, Belgium; ^5^Department of Cardiology, University Hospital UZ Leuven, Leuven, Belgium; ^6^Department of Thoracic and Cardiovascular Surgery, RWTH Aachen University Hospital, Aachen, Germany; ^7^Department of Cardiac Surgery, Hôpital Universitaire Pitié-Salpêtrière, Paris, France

**Keywords:** myocardial infarction, cardiogenic shock, VAD, mechanical support devices, Impella, ECMO, hemodynamics, heart assist devices

## Abstract

**Objectives:** Several mechanical circulatory assist devices are used to treat critically ill patients requiring hemodynamic support during post-myocardial infarction or cardiogenic shock. However, little guidance is available to choose an appropriate device to match a particular patient's needs. An increased understanding of hemodynamic effects of the pump systems and their impact on myocardial pre-/afterload might help to better understand their behavior in different clinical settings.

**Methods:** This was an open-labeled, randomized acute animal experiment. A model of acute univentricular myocardial injury by temporary balloon occlusion was used. The experiment was carried out in 10 juveniles female Piétrain pigs. The animals were randomized to mechanical hemodynamic support either by peripheral veno-arterial (VA-)ECMO or Impella CP.

**Results:** While both devices were able to provide flows above 3 L/min and maintain sufficient end-organ perfusion, support by Impella resulted in a significantly more pronounced immediate effect on myocardial unloading: At the onset of device support, the remaining native cardiac output was reduced by 23.5 ± 15.3% ECMO vs. 66.2 ± 36.2% (Impella, *p* = 0.021). Native stroke volume was significantly decreased by Impella support compared to ECMO, indicating less mechanical work being conducted by the Impella-supported hearts despite similar total assisted cardiac output.

**Conclusions:** Peripheral VA-ECMO and the transaortic Impella pump resulted in contrasting hemodynamic fingerprints. Both devices provided sufficient hemodynamic support and reduce left ventricular end-diastolic pressure in the acute setting. Treatment with the Impella device resulted in a more effective volume unloading of the left ventricle. A significant reduction in myocardial oxygen consumption equivalent was achieved by both devices: The Impella device resulted in a left-shift of the pressure-volume loop and a decreased pressure-volume-area (PVA), while VA-ECMO increased PVA but decreased heart rate. These data highlight the importance of specifically targeting heart rate in the management of AMI patients on hemodynamic support.

## Introduction

Several mechanical circulatory assist devices are currently used to treat critically ill patients who require hemodynamic support, such as during post-myocardial infarction (MI) or cardiogenic shock (CS). Each of these support platforms results in a device-specific effect on patient hemodynamics that can directly impact the patient, heightening the importance of an informed device choice (1). However, there is a paucity of data comparing the hemodynamics of these devices head-to-head in the same clinical conditions.

Based on the results from the IABP-SHOCK-II trial (2), ESC guidelines downgraded the Intra-aortic Balloon Pump (IABP) to a level III recommendation and thus discouraged the routine use of the IABP in CS. Indeed, the use of IABP in CS patients is declining (3). The use of peripheral veno-arterial extracorporeal membrane oxygenation (VA-ECMO) is on the rise despite a lack of evidence (4). Only few non-randomized studies demonstrated survival advantage of VA-ECMO use in CS (5). The current widespread acceptance and practice of VA-ECMO in CS patients is similar to the IABP's era prior to the IABP-SHOCK II trial (2). The invasiveness of VA-ECMO and the associated complications should not be neglected. VA-ECMO is often accompanied by an extended inflammatory response and severe side effects including limb ischemia, lower limb amputation, fasciotomy or compartment syndrome, stroke, and acute kidney injury (6). Elevated cardiac afterload, resulting from the retrograde aortic flow of the device, is a common observation in patients treated with peripheral VA-ECMO, (7) but it remains unclear to what extent this may exacerbate the underlying myocardial pathology. Several strategies for ventricular unloading during VA-ECMO support have been proposed (8).

The Impella pump (Abiomed Europe GmbH, Aachen, Germany), a percutaneous trans-aortic ventricular pump, offers a distinct alternative mechanical support device able to hemodynamically stabilize these critically ill patients. Unlike VA-ECMO, the Impella is a transvalvular pump with an inlet that resides within the left ventricle (LV) chamber. As such, it directly aspirates blood from the left ventricle and expels it into the aorta in parallel with native cardiac blood flow. Owing to this design, the Impella effectively unloads the LV while simultaneously stabilizing patient hemodynamics and augmenting cardiac output with up to 3.5 L/min (Impella CP) of blood flow, or above 5 L/min (Impella 5.0) (9). For this reason, the Impella pump is often deployed as an LV unloading strategy in VA-ECMO patients, resulting in improved patient hemodynamics (10).

Currently available clinical data on mechanical support devices does not indicate favor for any one device over another (11). To address this gap in knowledge, we use a porcine model of acute myocardial ischemia/reperfusion (I/R) injury to compare the hemodynamic differences between peripheral VA-ECMO and Impella CP ventricular support.

## Materials and Methods

**Figure 1:**
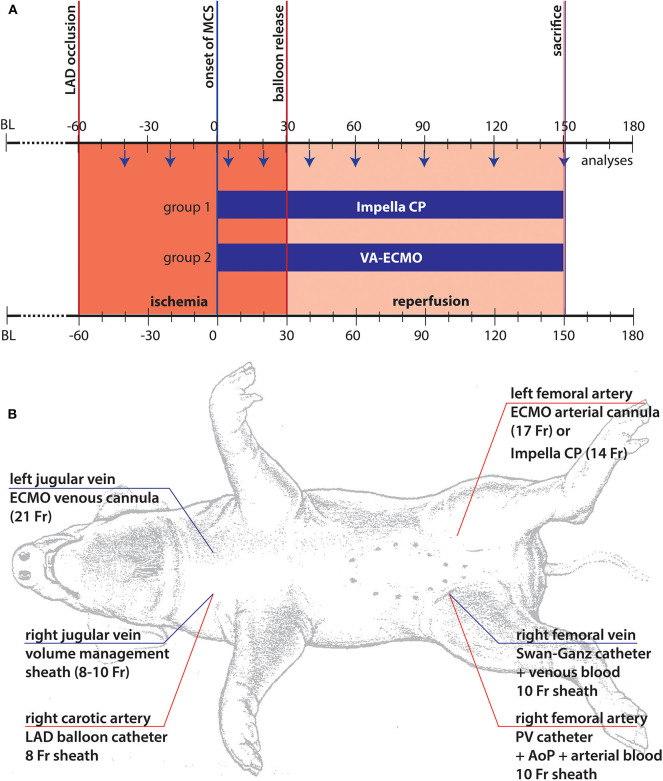
Illustration of experimental setup. **(A)** Timeline and experimental setup: The chosen model consisted of three different phases. In the first phase following coronary occlusion, marked in red, the animals were left in unsupported ischemia for 60 min. After this period, mechanical support was started (*t* = 0) following randomization and the animals were supported for another 30 min of ischemia by the respective device before reperfusion was initiated by re-opening of the coronary balloon. The animals were supported until sacrifice after 2 h of support under reperfusion; **(B)** The standardized animal instrumentation is shown. Of note, cannulation for MCS was only performed after randomization, thus, right sided cannulation depended on the chosen system of support.

All experiments were approved by the Leuven ethical board (approval no.014/2018). Experiments were carried out in 10 juveniles female Piétrain pigs, weighing 68.3 ± 8.14kg. After i.m. induction of general anesthesia (tiletamine/zolazepame – 8 mg/kg, xylazine hydrochloride – 2.5 mg/kg), anesthesia was maintained by continuous administration of propofol (10 mg/kg/h) and fentanyl (0.15 mg/kg/min). Animals were instrumented as shown in Figure 1B. For rhythm stabilization, animals were primed with 300 mg amiodarone and 0.5 mg/kg/h lidocaine was continuously administered (12). A protective mechanical ventilation, according to the Extracorporeal Life Support Organization (ELSO) guidelines (13), was maintained, including pressure controlled ventilation at an I:E ratio of 2:1, a respiratory rate of minimally 6/min with an FiO_2_ of 0.25, low tidal volume (6 ml/kg body weight) and high PEEP (≥ 10 cmH_2_O) while on ECMO. During the entire experiment, phenylephrine was the only vasoactive/inotropic medication allowed to maintain a mean arterial pressure above 50 mmHg. In case of ventricular fibrillation, external defibrillation was allowed to regain a stable rhythm.

Following the complete instrumentation of the animal (Figure 1B), baseline values were taken, and the protocol was carried out as demonstrated in Figure 1A. Briefly, following angiocardiographic determination of baseline ejection fraction (EF), the left anterior descending (LAD) coronary artery was wired and subsequently occluded by a 3.5 mm PTA balloon distal to the 2nd diagonal branch. Following 60 min of ischemia, either ECMO cannulas (21Fr venous, 17Fr arterial) or Impella (14Fr, Impella CP, Abiomed Europe GmbH, Aachen, Germany) were inserted and mechanical support was initiated at 3.5 L/min ECMO or maximum achievable unloading (Impella CP). After another 30 min on support, reperfusion was initiated by deflation of the balloon. Following 120 min of reperfusion, to assess infarct area at risk and infarcted myocardium the balloon was re-inflated followed by an intracoronary injection of 50 ml Evans blue (2%) per coronary vessel before the animal was euthanized by thiopental. The heart was then excised, thoroughly washed, and cut into equal slices of 1 cm thickness. Area at risk was determined as the unstained area of the LV prior to a 5 min staining with 1%TTC at 37°C to determine the infarction zone (13). During the period of pre-MCS support there were intrasubject variations, mainly due to the hemodynamic management required in this period of untreated myocardial ischemia. This often led to hemodynamic destabilization and arrythmia, requiring medical treatment. The model's intention was the generation of a comparable status prior to MCS initiation, achieved by stabilization of animals and weaning of vasopressors. This was successfully achieved in those animals retained in the study.

Heart rate, pressures in the aorta, LV, vena cava, pulmonary artery, and device flows as indicated by the device-specific consoles were continuously recorded throughout the experiment. At standardized intervals (Figure 1A) pressure volume loops to calculate left ventricular end systolic and end diastolic pressure and volume, stroke volume, native cardiac output and total pressure volume area were assessed. Blood samples were drawn at the same time points to measure arterial pH and lactate, carbon dioxide, creatine kinase (CK), troponin, hematocrit, hemoglobin, fibrinogen and plasma free hemoglobin in addition to central venous oxygen saturation.

### Statistical Analyses

Statistical evaluation was performed with SPSS-23 (IBM Corporation, Armonk, New York, USA). Figures were created using Graph Pad Prism version 7.0a for MAC OS X (Graph Pad Software, La Jolla California USA) and prepared for submission using Adobe® Illustrator® CS6 (Adobe Inc., San Jose, California, USA). The Kolmogorov-Smirnov test was used to assess the distribution of continuous variables. Normal distributed continuous variables are expressed as the means ± standard deviations (SDs) and non-normal distributed continuous variables as medians and Inter-quartile range (IQR). Categorical variables are expressed as absolute numbers and percentages. A mixed effects model was used for the comparison between the two groups to compare the intraindividual factor time, interindividual factor device and the combination of both. *P-*values generated from the mixed effects model were then adjusted with the Sidak's test for multiple comparisons. Comparison between different time points within each group was carried out with the non-parametric Friedman-test and followed by Dunn's correction for multiple comparisons. Additional comparison of hemodynamic parameters between and within the groups after exclusion of one extreme outlier in the Impella group (ECMO *n* = 5 vs. Impella *n* = 4) were performed and presented in the Supplementary File as Table S1 and Figure S1. Adjusted *p-*values for multiple comparison are presented. P < 0.05 was considered statistically significant (Asterisks: ^*^p < 0.05, ^**^p < 0.01, ^***^p < 0.001).

## Results

All supported animals survived until the end of the experiment. Baseline demographics did not differ significantly between groups: Body weight was 68 ± 6 kg in the Impella (I) group vs. 69 ± 11 kg in the ECMO (E, *p* = 0.86) group. Post-mortem analyses revealed a ventricular weight of 309 ± 28 g (I) vs. 304 ± 50 g (E, *p* = 0.85). Median Hb prior to instrumentation was 11.2 (2.2) g/dl (I) vs. 11.2 (1.80) g/dl (E, *p* = 0.85).

Animals suffered hemodynamic destabilization including all degrees of arrhythmias. This was especially pronounced during the unsupported ischemic phase. Due to episodes of ventricular fibrillation, short periods of mechanical CPR with defibrillations were required, and two animals were lost during this phase prior to randomization to a support group and were replaced. Pressor support could be weaned after re-stabilization of the animal in all cases.

AAR and infarct size were not different between the groups, indicating that extent of injury in both groups was the same. Infarct size as percent AAR was similar between groups (70.95 ± 10.64% vs. 74.63 ± 6.02%, *p* = 0.44).

### Hemodynamics

**Figure 2:**
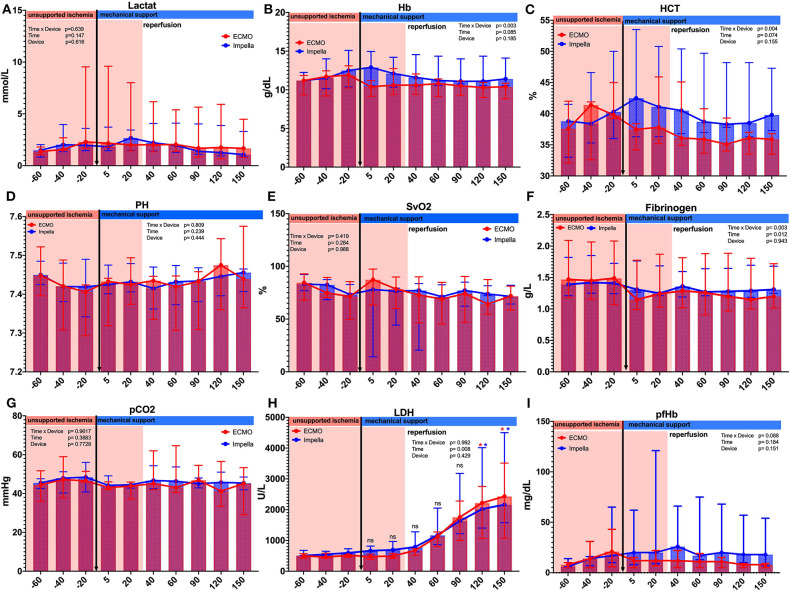
Comparison of laboratory parameter between and within the groups. Several laboratory parameters were captured during the experiment. **(A)** Lactate; **(B)** Hemoglobin g/dL;**(C)** Hematocrit %; **(D)** PH values; **(E)** Central venous saturation %; **(F)** Fibrinogen g/L; **(G)** Partial CO_2_ pressure mmHg; **(H)** Lactate dehydrogenase U/L; **(I)** Plasma free hemoglobin. Asterisks: *p < 0.05, black Asterisks indicates significance between the groups; blue Asterisks indicate significance within the Impella group compared to the time point *t* = −20; red Asterisks indicate significance within the ECMO group compared to the time point *t* = −20.

We observed the expected effects of hemodilution by priming volume in the ECMO-supported animals. Although not reaching statistical significance, we observed a numerical effect of hemodilution on hematocrit by priming volume in the ECMO-supported animals; blood parameters diverged after onset of MCS as visible in the hematocrit between groups (see Table 1 and Figure 2). As expected, both devices were able to supply sufficient end organ perfusion as assessed by normalization of serum lactate levels over time (Table 1 and Figure 2).

**Table 1:** Blood gas analyses and laboratory blood tests.

**Timepoint**		**Infarction**	**Pre-onset of**	**Onset of**	**Reperfusion**	**End of experiment**	*p-*value Time*device
		**(T-60)**	**MCS (t-20)**	**MCS (t5)**	**(t40)**	**(t150)**	**(device)**
**Parameter**	**Device**						
art. pH	Impella	7.40 (0.01)	7.41 (0.05)	7.42 (0.06)	7.41 (0.05)	7.45 (0.03)	0.315 (0.621)
	ECMO	7.41 (0.03)	7.40 (0.06)	7.43 (0.01)	7.43 (0.08)	7.43 (0.07)	
art. pCO2 (mmHg)	Impella	45.50 (3.2)	48.42 (5.8)	44.20 (2.60)	46.71 (4.2)	45.42 (4.0)	0.384 (0.569)
	ECMO	44.50 (8.70)	46.51 (2.10)	43.10 (2.10)	45.10 (3.20)	45.10 (6.40)	
venous cSo2 (%)	Impella	83.11 (7.59)	73.30 (7.59)	78 (2.20)	77.11 (7.59)	71.4 (8.09)	0.405 (0.497)
	ECMO	84.50 (3.19)	71.30 (16.40)	87.40 (14.60)	72.80 (29.70)	71.80 (14.20)	
art Lac (mmol/l)	Impella	1.69 (0.32)	2.59 (4.46)	3.46 (0.91)	2.65 (1.57)	2.27 (0.64)	0.414 (0.474)
	ECMO	1.73 (0.58)	2.88 (0.85)	2.7 (0.90)	2.88 (0.67)	1.68 (1.22)	
CK (U/L)	Impella	2538 (2504)	2623 (1554)	4533 (417)	7626 (6922)	20474 (5186)	0.861 (0.415)
	ECMO	1613 (1249)	2080 (1512)	3409 (770)	4833 (2509)	16048 (17380)	
Hematocrit (%)	Impella	38.8 (6.79)	40.3 (11.6)	42.5 (8.20)	40.5 (7.50)	39.8 (8.60)	0.018 (0.189)
	ECMO	37.6 (5.79)	39.8 (3.29)	36.2 (3.29)	36.1 (1.60)	34.7 (2.40)	
Hemoglobin (g/dl)	Impella	11.2 (2.2)	12.5 (4.1)	12.9 (2.4)	11.6 (2.7)	11.4 (3.1)	0.008 (0.289)
	ECMO	11.2 (1.80)	11.9 (1.11)	10.4 (0.69)	10.6 (0.60)	10.4 (0.89)	
Fibrinogen (g/l)	Impella	1.39 (0.38)	1.41 (0.22)	1.31 (0.33)	1.36 (0.21)	1.31 (0.30)	0.000 (0.719)
	ECMO	1.47 (0.65)	1.49 (0.70)	1.15 (0.52)	1.29 (0.57)	1.21 (0.51)	
pfHb (mg/dl)	Impella	6 (6)	17 (4)	20 (11)	26 (15)	18 (21)	0.318 (0.106)
	ECMO	8 (3)	21 (15)	12 (9)	12 (7)	8 (4)	

*Data are presented as median and inter-quartile range. pfHB, plasma free hemoglobin; CK, Creatinine kinase; venous cSo2, central venous saturation; art lac, arterial Lactate*.

**Figure 3:**
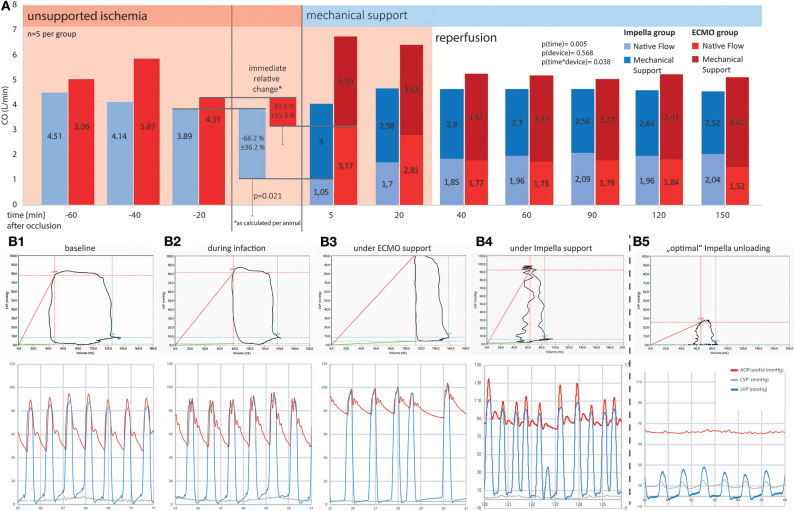
Comparison of cardiac output and pressure-volume loops between ECMO and Impella groups. Mechanical support is emphasized, showing a significantly more prominent reduction in native flow at the onset of Impella support as compared to ECMO. Ten minutes into the onset of reperfusion (*t* = 40 min and after), both systems were able to maintain a physiologic total (as the sum of native and device) output by providing between 2.5 and 3.5 L/min additional flow to the blood circuit. **(A)** Systemic flow as SV*HR (native equivalent) device flow over time **(A)** and as percentage of change on onset of device support; **(B)** Exemplary pressure-volume loops and corresponding pressure tracings for different timepoints in the experiment. **(B1)** Shows a baseline recording in a healthy animal. During myocardial infarction **(B2)**, the decrease in contractility leads to a reduction in left ventricular ejection fraction, the difference between diastolic and systolic LV volume decreases. ECMO **(B3)** is able to restore perfusion pressure but further diminishes ejection fraction. Without active unloading, the PV curve is right shifted to supra-normal volumes. **(B4,B5)** represent two different support situations under Impella treatment. While **(B4)** already shows a left shift of the PV loop, indicating ventricular volume unloading, the ventricle is still able to generate pressure and thus to contribute actively to blood expulsion in systole. **(B5)** Additionally shows uncoupling of the ventricular (blue) from the aortic pressure wave (red). This “optimal” unloading further minimizes the PV-area and thus the myocardial energy consumption as the myocardium is no longer actively contributing to blood flow. Especially in an unloaded (i.e., emptied) ventricle, co-location of the PV-catheter and the Impella pump causes signal interferences as demonstrated by the unstable lines in the PV-loop section of **B4/5**. Behold that while both devices reduce active ventricular ejection when supporting circulation, ECMO leads to a right shift and thus increases LV wall stress, whereas Impella left shifts the PV loop, thus reducing myocardial workload. For better visualization, in the lower graphs, arterial (red) and left ventricular (blue) pressure curves have been superimposed. Due to the distance in measurement, arterial signals are usually delayed by a notable offset.

The onset of MCS resulted in a notable increase in total cardiac output (native+pump) in both groups. While both devices were able to provide flows above 3 L/min, support by Impella resulted in a significant early effect on myocardial unloading: We noted an immediate reduction of native flow in the Impella group ([t-20] 3.9 ± 1.74 vs. [t5] 1.05 ± 1.24 L/min, *p* = 0.021) and the mean reduction achieved with Impella was 66.2 ± 36.2%. This effect diminished at later time points during the experiment. On the other hand, use of ECMO reduced the native flow only by 23.5 + 15.3% (4.3 ± 1.03 vs. 3.2 ± 0.53 L/min, *p* > 0.999). Further comparison at different time points are presented in Figure 3A. At later timepoints (> *t* = 5), both groups demonstrated statistically non-significant reduction in native flow with residual flows of about 2 l/min, thus about half of the initial native flow (Figure 3A). Thus, a reduction of active mechanical workload of the heart (native flow) was maintained in both groups for the duration of support after about 40 min of reperfusion. Total cardiac output as the sum of device and native flow did not differ significantly at any measured time points between devices (*p* = 0.108). Yet, a mere interpretation based on flow would omit the contribution of passive energy to the equation and pressure volume loop analyses are better suited to estimate the total myocardial energy consumption.

**Figure 4:**
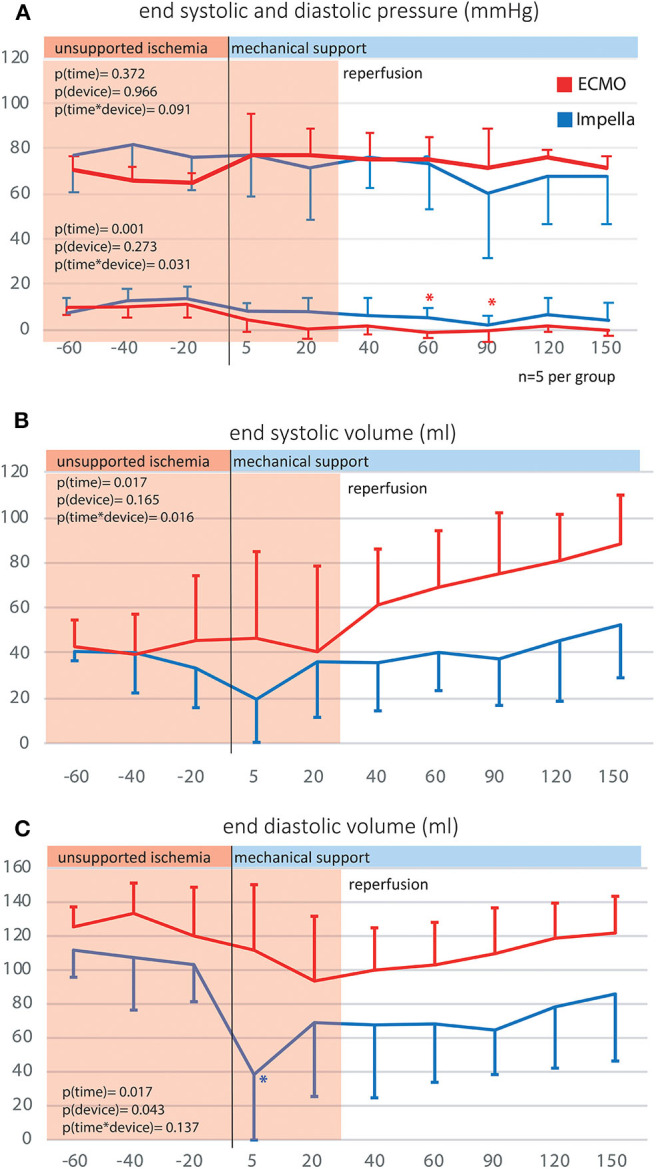
Left ventricular end systolic and diastolic volumes and pressures. By use of p-v catheters, the left intraventricular pressures and volumes were captured over time. **(A)** Left ventricular end systolic and diastolic pressure; **(B)** Left ventricular end systolic volume; **(C)** Left ventricular end diastolic volume.

**Figure 5:**
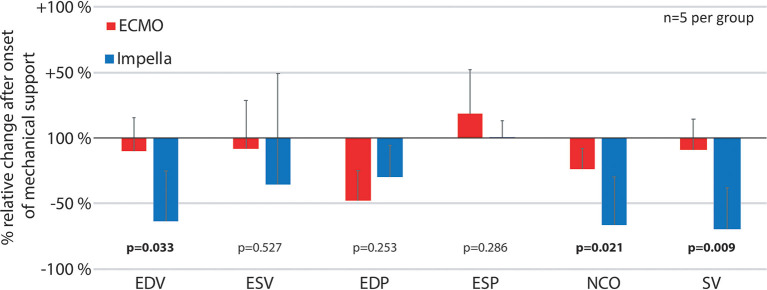
Relative pressure and volume differences after onset of mechanical support. The figure reflects the immediate changes in left ventricular pressures, volumes and native cardiac output as well as stroke volume by comparing the first time point after onset of mechanical support (*t* = 5) with the previous measurements (*t* = −20) as percentage of relative change for both devices. ESP, Left ventricular end systolic pressure; ESV, Left ventricular end systolic volume; EDP, end diastolic pressure; EDV, Left ventricular end diastolic volume; NCO, native cardiac output; SV, left ventricular stroke volume.

Both left ventricular pressures and volumes were analyzed by continuous PV loop measurement (Figure 3B). The Impella showed to be in principle able to completely unload the ventricle and decrease the mechanical workload of the heart in the setting of AMI (Figure 3B5). Yet, this maximal effectiveness was rarely achieved in our model, potentially due to suction or positioning issues. Figure 3B4 shows a typical PV loop of partial unloading. Complete unloading was not observed in ECMO-supported animals. Partial ventricular unloading as expressed by a significant reduction in LVEDP over time (*p* < 0.001) was achieved in both groups (Figure 4A) without a significant difference between devices (*p* = 0.414). Over time, ECMO lead to a substantial right shift of end-systolic volumes (Figure 4B). In the ECMO group, end-diastolic volumes were slightly reduced after onset of MCS yet returned back to baseline values until the end of the experiment [at *t* = −20 131 (50) mL vs. at *t* = 150 121(65) mL, *p* = 0.999]. For the Impella-treated animals, we detected a significant reduction in end-diastolic volume at onset of support compared to time point *t* = −20 [94 (15) mL at *t* = −20 vs. 31 (54) at *t* = 5, *p* = 0.032] and compared to ECMO group, EDV was significantly lower in the Impella group at t = 5 and *t* = 60 [31 (54) vs. 119 (73), *p* = 0.018 and 66 (19) vs. 102 (29) mL, *p* = 0.35, respectively]. In the Impella group, EDV staid reduced under Impella support and did not reach baseline values (*t* = −20) during the course of the experiment (Figure 4C). Importantly, stroke volume (SV) was significantly decreased by Impella support compared to ECMO (Figure 5 and Table 2), indicating less mechanical work-load to the Impella-supported hearts on a per beat basis despite similar total CO compared to the VA-ECMO group.

**Table 2:** Pressure and volume measurements.

**Timepoint**		**Baseline**	**Pre-onset of**	**Onset of**	**During reperfusion**	**End of experiment**	*p*-value time*device
		**(t-60)**	**MCS (t-20)**	**MCS (t5)**	**(t40)**	**(t150)**	**(device)**
**Parameter**	**Device**						
mAOP (mmHg)	Impella	65.8 ± 13.04	68.60 ± 12.47	73.6 ± 15.73	75.4 ± 14.88	70.8 ± 16.31	0.663 (0.335)
	ECMO	58.4 ± 3.07	51.20 ± 5.15	64.4 ± 15.86	67.4 ± 17.76	61.8 ± 5.04	
CVP (mmHg)	Impella	3 ± 2.1	4.40 ± 3.83	2.6 ± 2.73	3.2 ± 3.12	2.2 ± 2.48	0.082 (0.644)
	ECMO	2.2 ± 1.94	2.80 ± 2.48	0.4 ± 2.73	0.2 ± 2.04	1.4 ± 1.85	
mPAP (mmHg)	Impella	18.4 ± 1.36	23.20 ± 10.61	19.4 ± 5.61	21 ± 6.2	19.2 ± 4.02	0.005 (0.001)
	ECMO	17.2 ± 4.07	15.80 ± 3.71	11.2 ± 3.6	7.8 ± 1.6	6.6 ± 1.96	
SV (ml)	Impella	71 (24)	16 (24)	25 (19)	22 (34)	33 ± 24.22	0.003 (0.124)
	ECMO	80 (20)	66 (23)	41 (9)	33 (15)	33 ± 9.49	

*mAO, mean aortic pressure; CVP, central venous pressure; mPAP, mean pulmonary artery pressure*.

Due to the substantial reduction in right ventricular preload by ECMO, pulmonary artery pressures as measured by the pulmonary artery catheter are significantly reduced over time in sharp contrast to the Impella-treated animals (compare Table 2). Pulmonary flows are substantially reduced and can even be completely nullified by ECMO, but not by Impella (Figure S2). While one would expect this to result in a LV volume decrease over time, we observed the opposite in our animals.

To minimize the effect of physiological differences between animals, we also calculated the relative change at the onset of mechanical support for various pressure and volume variables (Figure 5). With increased ventricular unloading by Impella CP, the percent ΔEDV in this group was significantly more pronounced in the Impella group compared to VA-ECMO, which was also reflected by a significant decrease in stroke volume. As previously shown, there was a pronounced reduction of native cardiac output due to the addition of device flow (Figure 3A).

### Estimate of Myocardial Energetics

**Figure 6:**
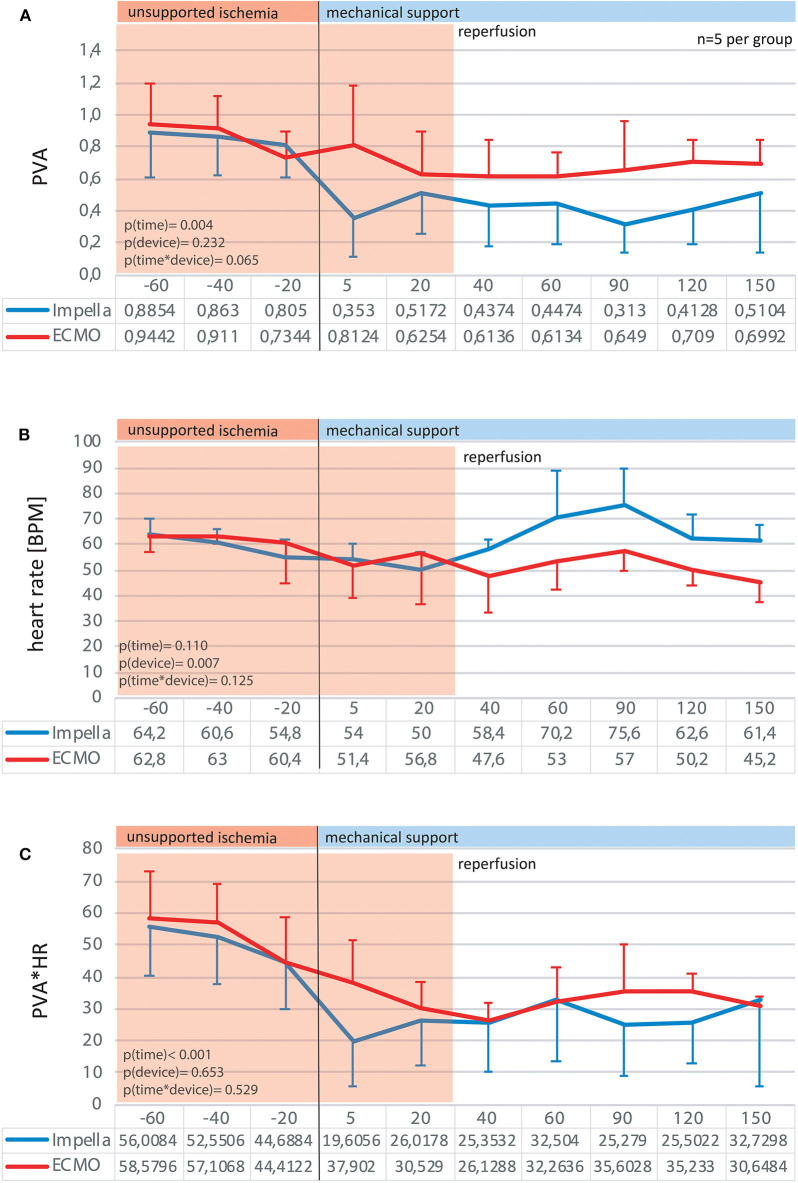
Myocardial energy consumption estimates. By analysis of the pressure-volume diagrams at given time point, the pressure-volume areas were traced as an estimate for myocardial work per beat **(A)**. Together with the heart rate **(B)** the product of pressure-volume area*heart rate can be used as a valid estimate for myocardial work-load over time **(C)**.

As myocardial oxygen consumption is known to correlate with the product of heart rate and pressure volume area, we estimated myocardial energetics by measuring HR, PVA and PVA^*^HR (Figure 6). While both devices reduced PVA significantly over time, the HR oscillated around baseline in the Impella group while a significant decrease in HR was notable for the VA-ECMO group. We observed no differences in total PVA^*^HR between the devices (Figure 6C). However, the determinant variable mediating this effect on PVA^*^HR varied between devices. The Impella group benefited most from its effect on reducing PVA, while the VA-ECMO group benefited most from its effect on reducing HR.

## Discussion

There were several new findings in the presented experiment: At the onset of device support, the native cardiac output was more substantially reduced by Impella as compared by ECMO, leading to a reduction in pressure volume area. Although we did not observe a significant difference in total PVA^*^HR between the devices, we must keep in mind that the HR as a determinant variable mediating this effect on PVA^*^HR varied between devices. In a setting of acute, predominantly left-ventricular MI with subsequent unloading and reperfusion, pressure-volume analyses showed substantial hemodynamic differences between two mechanical circulatory support strategies.

In the presented setting of left-dominant ventricular failure, both devices were able to provide sufficient hemodynamic support and improve end-organ perfusion. The Impella device initially unloaded the LV more effectively as measured by LVEDP, yet this effect was also reached by the peripheral ECMO after a support period of 40 min. Consistent with unloading and decreased ventricular work, the pressure-volume relationship in the Impella-supported hearts was left-shifted and the pressure-volume area was also decreased. In the ECMO group, ventricular unloading was achieved by preload diminution, i.e., reduction in pulmonary flow and pressure. In addition, heart rate was lower in ECMO as compared to Impella-treated animals.

Peripheral VA-ECMO was able to restore systemic perfusion, but it simultaneously led to an increase in diastolic volume and a right shift of PV-loops. Direct ventricular unloading by the Impella CP resulted in a reduced EDV, stroke volume, and PVA. These data indicate that compared to peripheral VA-ECMO, Impella support during left-sided AMI may be more effective at ventricular unloading. Indeed, recent data from Watanabe et al. (13) demonstrate that unloading by Impella decreased LV end-diastolic wall stress and significantly increased microvascular perfusion in the infarct zone significantly. Effective LV unloading and the resultant decrease in wall stress has direct clinical implications in the setting of AMI, where it predicts post-discharge incidence of HF (14).

An important pathophysiology of peripheral VA-ECMO is the so called “watershed phenomenon” (15): retrograde oxygenated ECMO flow meets and competes with the antegrade deoxygenated LV stroke volume at a point called “watershed point.” The watershed point can be located somewhere between the aortic root and the renal arteries. The lower the LV output compared to the ECMO flow, the more proximal the watershed point. This phenomenon can thus lead to perfusion of coronaries and first branches of the aortic arch with deoxygenated blood from the LV (16, 17). This being said, ECMO retrograde flow can even overcome LV intrinsic stroke volume and prevent aortic valve opening, especially in an LV with severe reduced contractility, leading to blood stasis, LV distension and increased LV wall stress, all these conditions being fatal (5, 9, 14, 17). While increasing ECMO-flow by emptying of the RV system will also subsequently reduce LV preload and might therefore reduce LV end-diastolic pressure and wall stress, the opposite effect might be promoted by the sharp increase in afterload (4, 5). The existence of physiological direct blood return to the LA via inter alia Thebesian veins and bronchial veins, increasing the ECMO's flow could even prohibit the described decrease in LV preload, and further aggravate LV distension and wall stress (4, 5, 7, 17).

Thus, finding optimal ECMO setting for each patient may be extremely important for achieving LV unloading using ECMO.

These data highlight the importance of fully understanding both patient hemodynamics and the hemodynamics consequences of specific support devices. HR is a major contributor to myocardial oxygen consumption. This can be appreciated when considering the classic formula for CO, *CO* = HR^*^SV. As SV equals essentially the mechanical work exerted by the heart while pumping blood, the energy required for conducting this work can be estimated by the PVA on a per beat basis. As Sagawa et al. (18) described, myocardial oxygen consumption can be described as a correlation of PVA^*^HR or as Tanaka et al. (19) described it in their canine setup, under constant PVA, HR, and mVO2 showed a good linear positive correlation (*r* = 0.824–0.995). Thus, within the experimental setup used, PVA^*^HR was utilized as an estimate on myocardial oxygen consumption. During Mechanical support there was a wide variation in HR within and between the groups, thus in our setting PVA^*^HR did not differ between Impella and ECMO groups. In our experiment, we did not control the HR. The relevance of HR reduction in the setting of myocardial infarction was recently described by Sunagawa et al. (20) in their publication demonstrating the synergistic effect of Impella mechanical circulatory support and simultaneous heart rate reduction. In a canine model of MI, Impella alone was able to reduce myocardial infarction by roughly 30%. Importantly, this effect was boosted to more than 55% when ivabradine was added as a bradycardic agent. Together with our new data, this suggests that targeting heart rate while on Impella support may further increase its effect on MVO_2_ sparing and promote better outcomes.

It is interesting to note that HR was lower in the VA-ECMO treated animals despite these animals having lower aortic pressure. These data go against what is expected from the classic baroreceptor response in which low aortic pressure triggers a compensatory increase in heart rate. While speculative, this suggests that the effect of VA-ECMO on decreasing HR was independent of aortic pressure, and may involve an intrinsic effect on the heart itself such as the Bezold-Jarisch reflex response and alterations of the coronary blood flow (21). Further investigations are required to prove this hypothesis.

It is important to further consider device-specific hemodynamic consequences. Chief amongst these may be the differential effect of VA-ECMO and Impella support on pulmonary circulation. While both devices are able to maintain sufficient cardiac output and perfusion pressure while unloading the heart, this is at the expense of considerably different device-specific hemodynamics. The Impella pumps blood in parallel with the heart. It aspirates blood directly from the LV into the aorta, thereby maintaining physiological flow. On the other hand, while VA-ECMO also pumps in parallel with heart, it results in a non-physiological flow. On VA-ECMO, blood is extracted from the femoral vein and infused into the femoral artery. This leads to complete by-pass of the lungs and heart (or nearly complete, depending on the level of support). The subsequent decrease in pulmonary artery pressure places the lungs at an acutely elevated risk for ischemia, which has since long been recognized as an inherent complication of VA-ECMO (22). Within the setting of AMI, this risk is expected to be exacerbated. Furthermore, combining these direct effects of ECMO on pulmonary circulation with its effect on LV distention and increased LV afterload (as observed here) could potentially compound complications leading to subsequent pulmonary edema. For this reason, prolonged use of VA-ECMO, especially in patients with depressed cardiac function often results in the need to mechanically unload, or vent, the LV by alternative means to alleviate these risks such as the Impella (7, 23–25).

## Limitations

Coronary artery disease is often progressive in humans and can lead to chronic ischemic conditions, forcing the heart to structurally and/or electrically remodel. This altered physiology is completely missing in juvenile animal models. No evidence regarding functional long-term outcomes was gathered. Nevertheless, the monitored increase in ventricular volumes over time during ECMO support may be interpreted as a precursor for a progressive ventricular congestion that is only compensated as long as the heart is still actively ejecting and might be lost in pre-diseased patients.

This was a model of univentricular failure. While VA-ECMO shows a broader range of use, also extending to biventricular failure, it has been shown that parallel unloading of the LV by Impella can prevent LV distension and improve outcome (25, 26).

## Conclusions

Peripheral VA-ECMO and the transaortic Impella pump resulted in contrasting hemodynamic fingerprints in a model of left coronary occlusion, resulting in a predominantly left-dominant CS. At the onset of device support, the native cardiac output was reduced by 23.5 ± 15.3% ECMO vs. 66.2 ± 36.2% (Impella). While both devices were able to provide sufficient hemodynamic support and reduce left ventricular end-diastolic pressure up to 3.5 h, treatment with the Impella device resulted in a more effective volume unloading of the left ventricle. The Impella device resulted in a left-shift of the pressure-volume loop and a decreased PVA, while peripheral VA-ECMO increased PVA but decreased HR. These data highlight the clinical relevance of specifically targeting HR in the management of AMI patients on hemodynamic support.

## Data Availability Statement

The raw data supporting the conclusions of this article will be made available by the authors, without undue reservation, to any qualified researcher.

## Ethics Statement

The animal study was reviewed and approved by Leuven ethical board approval no.014/2018.

## Author Contributions

CN, KI, BM, PL, and GL contributed to the conception and design of the study and contributed to the interpretation of the results. CN, KI, SY, SB, TA, and GL carried out the animal experiments. CN, SY, and SB contributed to sample preparation. CN and SB collected the data. CN, RZ, and SB organized the database. CN and RZ performed the statistical analysis. CN wrote the first draft of the manuscript. All authors provided critical feedback and helped shape the research, analysis and manuscript. All authors read and approved the submitted version.

## Conflict of Interest

The authors declare that this study received funding from Abiomed Europe GmbH. The funder had the following involvement with the study: full financial support of the entire series. CN and SB are full time employees of Abiomed Europe GmbH.

## Supplementary Material

Figure S1 Time course of native cardiac output, total cardiac output, left ventricular end diastolic volume and end systolic volume without an extreme outlier in the Impella Group (ECMO *n* = 5 vs. Impella *n* = 4).

Figure S2 Pulmonary flow as percentage of total flow in an open chest sheep model. As no direct flow measurement was used during the presented animal series, the presented data is from a preceding series of 5 sheep per group, undergoing the same protocol in an open chest setting where a flow probe was attached to the pulmonary trunk. Normalized to baseline, the bars clearly show the different effect of ECMO vs. Impella on pulmonary flow, up to an effective nullification of flows in 4/5 animals after 120 min of support.

Table S1 Comparison of hemodynamic between and within the groups. This table presents the hemodynamic data after exclusion of an extreme outlier as measured by infarct size in the Impella Group (ECMO *n* = 5 vs. Impella *n* = 4).
