# Supplementary figures and images for: Comparison of Hemodynamic Support by Impella vs. Peripheral Extra-Corporeal Membrane Oxygenation: A Porcine Model of Acute Myocardial Infarction
Source: Front Cardiovasc Med. 2020 Jun 10;7:99. doi: 10.3389/fcvm.2020.00099 (PMC7299088; doi:10.3389/fcvm.2020.00099)

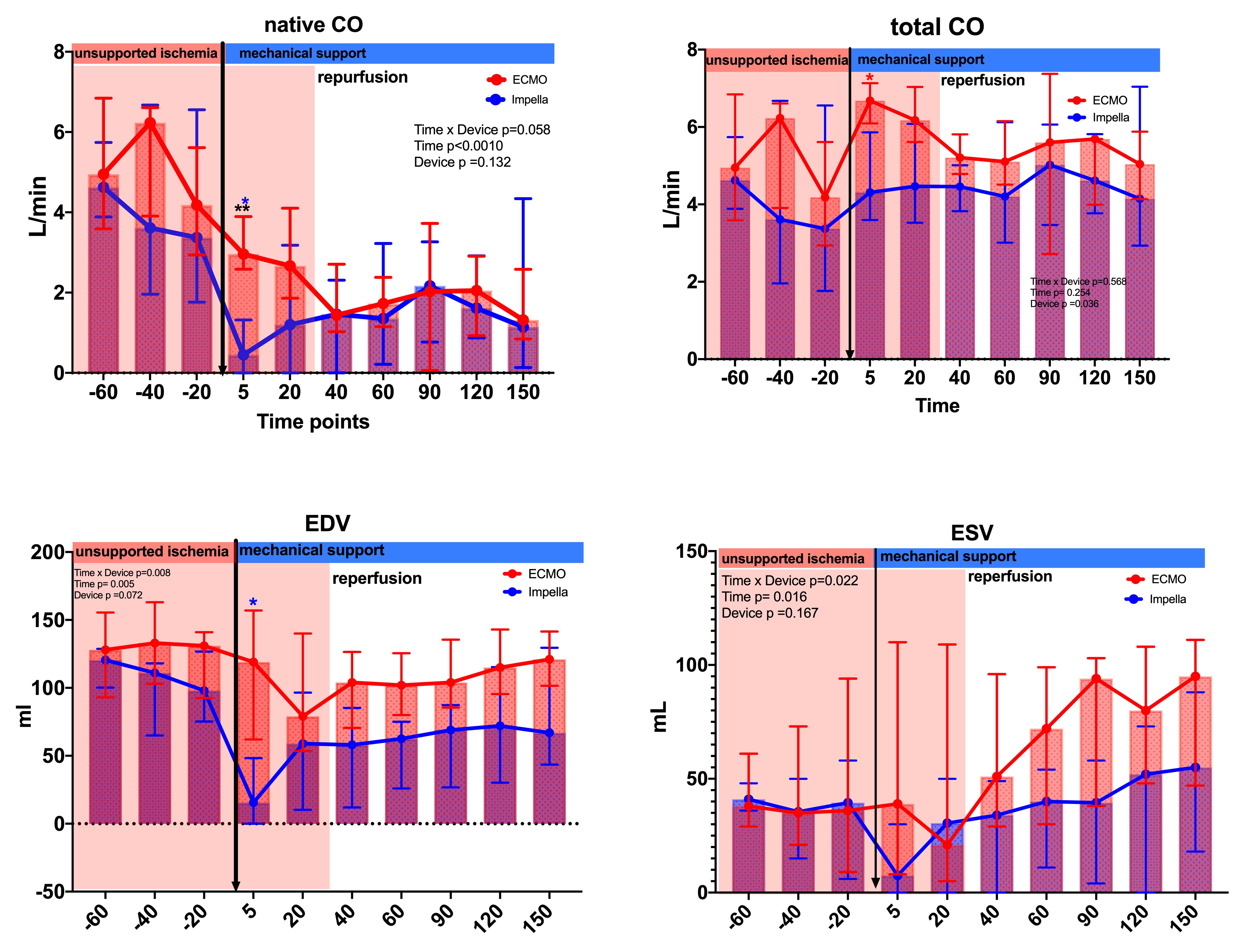

Supplement: Figure S1 — Time course of native cardiac output, total cardiac output, left ventricular end diastolic volume and end systolic volume without an extreme outlier in the Impella Group (ECMO n = 5 vs. Impella n = 4). [file Image_1.JPEG]

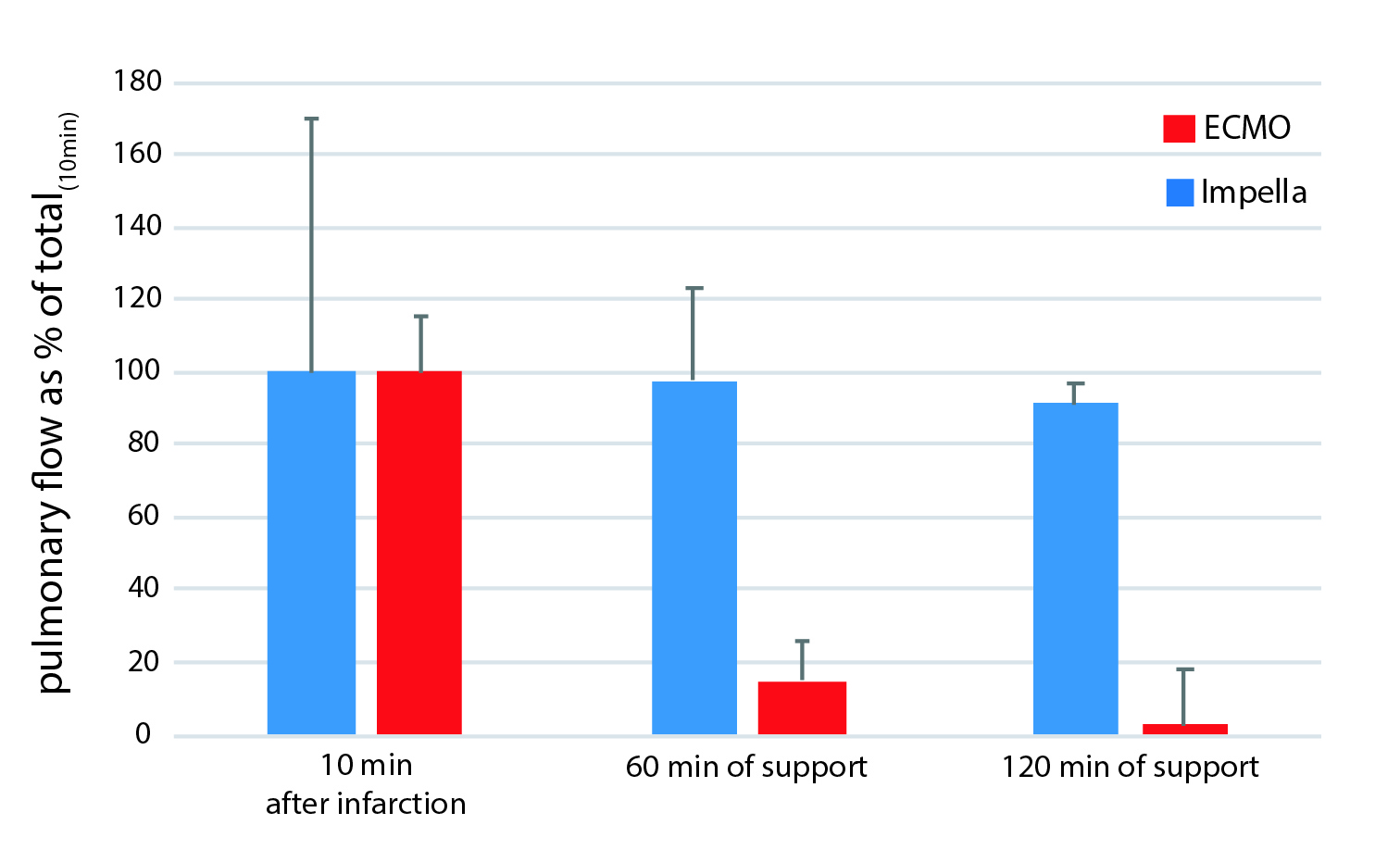

Supplement: Figure S2 — Pulmonary flow as percentage of total flow in an open chest sheep model. As no direct flow measurement was used during the presented animal series, the presented data is from a preceding series of 5 sheep per group, undergoing the same protocol in an open chest setting where a flow probe was attached to the pulmonary trunk. Normalized to baseline, the bars clearly show the different effect of ECMO vs. Impella on pulmonary flow, up to an effective nullification of flows in 4/5 animals after 120 min of support. [file Image_2.JPEG]
