# Supplementary material for: Comparison of Hemodynamic Support by Impella vs. Peripheral Extra-Corporeal Membrane Oxygenation: A Porcine Model of Acute Myocardial Infarction
Source: Front Cardiovasc Med. 2020 Jun 10;7:99. doi: 10.3389/fcvm.2020.00099 (PMC7299088; doi:10.3389/fcvm.2020.00099)
Supplement: Table S1 — Comparison of hemodynamic between and within the groups. This table presents the hemodynamic data after exclusion of an extreme outlier as measured by infarct size in the Impella Group (ECMO n = 5 vs. Impella n = 4). [file Table_1.DOCX]

Supplementary File:

**Table S1: Comparison of hemodynamic between and within the groups after exclusion of the Outlier in the Impella Group:**

| Native cardiac output |  |  |  |  |
| --- | --- | --- | --- | --- |
| Comparison of different time points Within each group | | | | |
| Dunn's multiple comparisons test | Mean rank diff. | Significant? | Summary | Adjusted P Value |
| ET -20 vs. ET5 | 9.000 | No | ns | >0.9999 |
| ET -20 vs. ET20 | 16.00 | No | ns | >0.9999 |
| ET -20 vs. ET40 | 33.70 | No | ns | 0.2214 |
| ET -20 vs. ET60 | 34.60 | No | ns | 0.1852 |
| ET -20 vs. ET90 | 34.00 | No | ns | 0.2087 |
| ET -20 vs. ET120 | 33.40 | No | ns | 0.2348 |
| ET -20 vs. ET150 | 39.90 | No | ns | 0.0599 |
| IT -20 vs. IT5 | 47.88 | Yes | * | 0.0304 |
| IT -20 vs. IT20 | 32.75 | No | ns | 0.5033 |
| IT -20 vs. IT40 | 32.38 | No | ns | 0.5338 |
| IT -20 vs. IT60 | 30.50 | No | ns | 0.7108 |
| IT -20 vs. IT90 | 20.25 | No | ns | >0.9999 |
| IT -20 vs. IT120 | 26.75 | No | ns | >0.9999 |
| IT -20 vs. IT150 | 30.00 | No | ns | 0.7656 |
| Native CO: comparison Between the groups | | | | |
| Sidak's multiple comparisons test | Mean Diff. | 95.00% CI of diff. | Summary | Adjusted P Value |
|  |  |  |  |  |
| ECMO - Impella |  |  |  |  |
| -20 | 0.545 | -5.099 to 6.190 | ns | >0.9999 |
| 5 | 2.616 | 0.8253 to 4.407 | ** | 0.0080 |
| 20 | 1.417 | -2.275 to 5.109 | ns | 0.7310 |
| 40 | 0.464 | -2.147 to 3.075 | ns | 0.9975 |
| 60 | 0.1985 | -4.060 to 4.457 | ns | >0.9999 |
| 90 | -0.301 | -3.762 to 3.158 | ns | >0.9999 |
| 120 | 0.0866 | -2.460 to 2.633 | ns | >0.9999 |
| 150 | -0.172 | -5.940 to 5.595 | ns | >0.9999 |
| Total Cardiac output |  |  |  |  |
| Comparison between groups | | | | |
| Sidak's multiple comparisons test | Mean Diff. | 95.00% CI of diff. | Summary | Adjusted P Value |
|  |  |  |  |  |
| ECMO - Impella |  |  |  |  |
| -60 | 0.5568 | -2.151 to 3.265 | ns | 0.9974 |
| -40 | 1.728 | -2.038 to 5.493 | ns | 0.6736 |
| -20 | 0.5452 | -5.099 to 6.190 | ns | >0.9999 |
| 5 | 2.129 | -0.7713 to 5.030 | ns | 0.1390 |
| 20 | 1.667 | -1.461 to 4.796 | ns | 0.3583 |
| 40 | 0.7829 | -0.5304 to 2.096 | ns | 0.3565 |
| 60 | 0.8305 | -3.408 to 5.069 | ns | 0.9817 |
| 90 | 0.2441 | -3.643 to 4.131 | ns | >0.9999 |
| 120 | 0.5126 | -2.006 to 3.032 | ns | 0.9948 |
| 150 | 0.3341 | -4.996 to 5.665 | ns | >0.9999 |
| Total Cardiac output |  |  |  |  |
| Comparison of different time points Within each group | | | | |
| Dunn's multiple comparisons test | Mean rank diff. | Significant? | Summary | Adjusted P Value |
| ET -20 vs. ET5 | -41.10 | Yes | * | 0.0266 |
| ET -20 vs. ET20 | -36.60 | No | ns | 0.0797 |
| ET -20 vs. ET40 | -15.50 | No | ns | >0.9999 |
| ET -20 vs. ET60 | -16.10 | No | ns | >0.9999 |
| ET -20 vs. ET90 | -13.80 | No | ns | >0.9999 |
| ET -20 vs. ET120 | -15.90 | No | ns | >0.9999 |
| ET -20 vs. ET150 | -9.500 | No | ns | >0.9999 |
| IT -20 vs. IT5 | -7.875 | No | ns | >0.9999 |
| IT -20 vs. IT20 | -10.25 | No | ns | >0.9999 |
| IT -20 vs. IT40 | -5.500 | No | ns | >0.9999 |
| IT -20 vs. IT60 | -6.750 | No | ns | >0.9999 |
| IT -20 vs. IT90 | -14.50 | No | ns | >0.9999 |
| IT -20 vs. IT120 | -10.50 | No | ns | >0.9999 |
| IT -20 vs. IT150 | -7.000 | No | ns | >0.9999 |
| End systolic pressure |  |  |  |  |
| Comparison of different time points Within each group | | | | |
|  |  |  |  |  |
| Dunn's multiple comparisons test | Mean rank diff. | Significant? | Summary | Adjusted P Value |
| ET -20 vs. ET5 | -16.50 | No | ns | >0.9999 |
| ET -20 vs. ET20 | -18.60 | No | ns | >0.9999 |
| ET -20 vs. ET40 | -13.90 | No | ns | >0.9999 |
| ET -20 vs. ET60 | -17.30 | No | ns | >0.9999 |
| ET -20 vs. ET90 | -10.50 | No | ns | >0.9999 |
| ET -20 vs. ET120 | -20.30 | No | ns | >0.9999 |
| ET -20 vs. ET150 | -12.20 | No | ns | >0.9999 |
| IT -20 vs. IT5 | -0.8750 | No | ns | >0.9999 |
| IT -20 vs. IT20 | 4.125 | No | ns | >0.9999 |
| IT -20 vs. IT40 | 4.125 | No | ns | >0.9999 |
| IT -20 vs. IT60 | 1.750 | No | ns | >0.9999 |
| IT -20 vs. IT90 | 0.6250 | No | ns | >0.9999 |
| IT -20 vs. IT120 | 3.500 | No | ns | >0.9999 |
| IT -20 vs. IT150 | 5.375 | No | ns | >0.9999 |
| End systolic pressure |  |  |  |  |
| Between groups |  |  |  |  |
| ECMO - Impella |  |  |  |  |
| -60 | -8.10 | -74.46 to 58.26 | ns | 0.9989 |
| -40 | -16.8 | -81.40 to 47.80 | ns | 0.8735 |
| -20 | -13.75 | -70.86 to 43.36 | ns | 0.9041 |
| 5 | -0.700 | -64.61 to 63.21 | ns | >0.9999 |
| 20 | 3.100 | -89.87 to 96.07 | ns | >0.9999 |
| 40 | 0.8000 | -45.61 to 47.21 | ns | >0.9999 |
| 60 | 3.100 | -76.25 to 82.45 | ns | >0.9999 |
| 90 | -0.60 | -66.27 to 65.07 | ns | >0.9999 |
| 120 | 4.650 | -86.99 to 96.29 | ns | >0.9999 |
| 150 | -1.80 | -82.29 to 78.69 | ns | >0.9999 |
| End-diastolic pressure |  |  |  |  |
| Within each group |  |  |  |  |
| Dunn's multiple comparisons test | Mean rank diff. | Significant? | Summary | Adjusted P Value |
| ET -20 vs. ET5 | 19.20 | No | ns | >0.9999 |
| ET -20 vs. ET20 | 33.50 | No | ns | 0.1565 |
| ET -20 vs. ET40 | 27.90 | No | ns | 0.4845 |
| ET -20 vs. ET60 | 39.10 | Yes | * | 0.0429 |
| ET -20 vs. ET90 | 36.60 | No | ns | 0.0781 |
| ET -20 vs. ET120 | 27.20 | No | ns | 0.5517 |
| ET -20 vs. ET150 | 34.70 | No | ns | 0.1203 |
| IT -20 vs. IT5 | 7.500 | No | ns | >0.9999 |
| IT -20 vs. IT20 | 11.00 | No | ns | >0.9999 |
| IT -20 vs. IT40 | 21.75 | No | ns | >0.9999 |
| IT -20 vs. IT60 | 18.50 | No | ns | >0.9999 |
| IT -20 vs. IT90 | 19.25 | No | ns | >0.9999 |
| IT -20 vs. IT120 | 11.13 | No | ns | >0.9999 |
| IT -20 vs. IT150 | 31.13 | No | ns | 0.4901 |
| End-diastolic pressure |  |  |  |  |
| Between groups |  |  |  |  |
| Dunn's multiple comparisons test | Mean rank diff. | Significant? | Summary | Adjusted P Value |
| ET -20 vs. ET5 | -16.50 | No | ns | >0.9999 |
| ET -20 vs. ET20 | -18.60 | No | ns | >0.9999 |
| ET -20 vs. ET40 | -13.90 | No | ns | >0.9999 |
| ET -20 vs. ET60 | -17.30 | No | ns | >0.9999 |
| ET -20 vs. ET90 | -10.50 | No | ns | >0.9999 |
| ET -20 vs. ET120 | -20.30 | No | ns | >0.9999 |
| ET -20 vs. ET150 | -12.20 | No | ns | >0.9999 |
| IT -20 vs. IT5 | -0.875 | No | ns | >0.9999 |
| IT -20 vs. IT20 | 4.125 | No | ns | >0.9999 |
| IT -20 vs. IT40 | 4.125 | No | ns | >0.9999 |
| IT -20 vs. IT60 | 1.750 | No | ns | >0.9999 |
| IT -20 vs. IT90 | 0.6250 | No | ns | >0.9999 |
| IT -20 vs. IT120 | 3.500 | No | ns | >0.9999 |
| IT -20 vs. IT150 | 5.375 | No | ns | >0.9999 |
| End-diastolic volume |  |  |  |  |
| Within groups |  |  |  |  |
| Dunn's multiple comparisons test | Mean rank diff. | Significant? | Summary | Adjusted P Value |
| ET -20 vs. ET5 | 7.500 | No | ns | >0.9999 |
| ET -20 vs. ET20 | 16.00 | No | ns | >0.9999 |
| ET -20 vs. ET40 | 11.20 | No | ns | >0.9999 |
| ET -20 vs. ET60 | 10.10 | No | ns | >0.9999 |
| ET -20 vs. ET90 | 6.200 | No | ns | >0.9999 |
| ET -20 vs. ET120 | 1.100 | No | ns | >0.9999 |
| ET -20 vs. ET150 | 0.3000 | No | ns | >0.9999 |
| IT -20 vs. IT5 | 36.00 | No | ns | 0.2095 |
| IT -20 vs. IT20 | 20.88 | No | ns | >0.9999 |
| IT -20 vs. IT40 | 24.63 | No | ns | >0.9999 |
| IT -20 vs. IT60 | 25.50 | No | ns | >0.9999 |
| IT -20 vs. IT90 | 21.00 | No | ns | >0.9999 |
| IT -20 vs. IT120 | 14.13 | No | ns | >0.9999 |
| IT -20 vs. IT150 | 13.38 | No | ns | >0.9999 |
| End-diastolic volume |  |  |  |  |
| Between groups |  |  |  |  |
| Sidak's multiple comparisons test | Mean Diff. | 95.00% CI of diff. | Summary | Adjusted P Value |
|  |  |  |  |  |
| Impella - ECMO |  |  |  |  |
| -20 | -19.60 | -90.39 to 51.19 | ns | 0.9471 |
| 5 | -90.15 | -192.4 to 12.08 | ns | 0.0874 |
| 20 | -37.95 | -159.2 to 83.29 | ns | 0.9147 |
| 40 | -47.85 | -147.9 to 52.20 | ns | 0.5266 |
| 60 | -48.10 | -119.5 to 23.35 | ns | 0.2419 |
| 90 | -48.20 | -135.3 to 38.87 | ns | 0.4360 |
| 120 | -45.90 | -164.1 to 72.30 | ns | 0.6714 |
| 150 | -41.40 | -178.1 to 95.27 | ns | 0.8199 |
| End-systolic volume |  |  |  |  |
| Between groups |  |  |  |  |
| Sidak's multiple comparisons test | Mean Diff. | 95.00% CI of diff. | Summary | Adjusted P Value |
|  |  |  |  |  |
| ECMO - Impella |  |  |  |  |
| -60 | 1.500 | -27.63 to 30.63 | ns | >0.9999 |
| -40 | 5.600 | -42.87 to 54.07 | ns | >0.9999 |
| -20 | 9.850 | -62.94 to 82.64 | ns | 0.9999 |
| 5 | 35.35 | -61.38 to 132.1 | ns | 0.7912 |
| 20 | 12.85 | -80.35 to 106.1 | ns | 0.9999 |
| 40 | 32.15 | -35.21 to 99.51 | ns | 0.6392 |
| 60 | 32.95 | -30.50 to 96.40 | ns | 0.5406 |
| 90 | 39.95 | -35.45 to 115.4 | ns | 0.5194 |
| 120 | 36.75 | -57.94 to 131.4 | ns | 0.7389 |
| 150 | 34.40 | -46.60 to 115.4 | ns | 0.7024 |
| End-systolic volume |  |  |  |  |
| Within groups |  |  |  |  |
|  |  |  |  |  |
| Dunn's multiple comparisons test | Mean rank diff. | Significant? | Summary | Adjusted P Value |
| ET -20 vs. ET5 | -0.3000 | No | ns | >0.9999 |
| ET -20 vs. ET20 | 3.500 | No | ns | >0.9999 |
| ET -20 vs. ET40 | -10.00 | No | ns | >0.9999 |
| ET -20 vs. ET60 | -14.80 | No | ns | >0.9999 |
| ET -20 vs. ET90 | -17.00 | No | ns | >0.9999 |
| ET -20 vs. ET120 | -22.60 | No | ns | >0.9999 |
| ET -20 vs. ET150 | -25.40 | No | ns | 0.7685 |
| IT -20 vs. IT5 | 18.00 | No | ns | >0.9999 |
| IT -20 vs. IT20 | 5.750 | No | ns | >0.9999 |
| IT -20 vs. IT40 | 5.250 | No | ns | >0.9999 |
| IT -20 vs. IT60 | -0.2500 | No | ns | >0.9999 |
| IT -20 vs. IT90 | -0.8750 | No | ns | >0.9999 |
| IT -20 vs. IT120 | -4.750 | No | ns | >0.9999 |
| IT -20 vs. IT150 | -9.875 | No | ns | >0.9999 |

Comparison of different time points to baseline (t-20) within each group was performed using the Kruskal-Wallis non-parametric test followed by Dunn’s correction for multiple comparison. Between groups analysis was performed with mixed-effecst model followed by Sidak’s correction for multiple comparisons.

E: ECMO, I: Impella
